# Honors to American Dentists

**Published:** 1853-10

**Authors:** 


*Honors to American Dentists.*—We recently had the pleasure of meeting Dr. Evans, surgeon dentist to the present ruler of France, who has, in the course of the past six years, attained a very high reputation. He is regarded as the first dentist on the continent of Europe, and superintends more regal and imperial teeth than all the rest of them put together, to say nothing of ducal and other lower grades of aristocratic masticators.

These "porcelain" gentry have one very attractive quality as customers. They generally are more substantially grateful than the commoner specimens of human pottery. Dr. Evans has several very beautiful tokens of regard from these distinguished individuals.

Louis Napoleon has given him an elegant gold jewel-box, richly set with diamonds in cyphers and N's, and placed on a pedestal of green velvet, adorned with golden bees.

The Queen of Holland has given him a still more costly box of the same character, most richly enamelled and studded with larger diamonds.

Add to these an opal breast-pin from Austria; a set of agate vest-buttons, ornamented with rubies and diamonds, from Baden Baden; a jeweled ring from that noble, heroic princess, the exiled Duchess of Orleans; the cross of the legion of honor from Louis Napoleon; with a host of similar trifles from various notabilities, and our readers will have an idea of what a very pleasant business it is to relieve the minor troubles of the magnificoes.

We must say for Dr. Evans, that he bears his honors very modestly, and that he is amiable, gentlemanly, aud unaffected in his deportment. Some envious curs will, of course, snarl at him, and say ill-natured things of him for exhibiting these things, but their spite is occasioned by his *hav-*

*ing* the mementoes and not by his *showing* them. For our own part, we think it a very commendable and honest pride, which induces a man to exhibit the trophies of his own skill, industry, and application to business. Besides this, a prudent man, who wishes to increase his business, will do this very thing, knowing, as he does, that there are multitudes of snobs who would pay any thing to have the same hand introduced into their vulgar mouths, that has wandered over the portals of an emperor's throat. The silly frogs that bite at red flannel are not without their analogues among more intelligent animals.

